# nim gene-independent metronidazole-resistant Bacteroides fragilis in surgical site infections

**DOI:** 10.3205/dgkh000298

**Published:** 2017-08-17

**Authors:** Mohammad Taghi Akhi, Reza Ghotaslou, Naser Alizadeh, Mina Yekani, Samad Beheshtirouy, Mohammad Asgharzadeh, Tahereh Pirzadeh, Mohammad Yousef Memar

**Affiliations:** 1 Research Center of Infectious and Tropical Disease, Tabriz University of Medical Sciences, Tabriz, Iran; 2 Department of Bacteriology and Virology, School of Medicine, Tabriz University of Medical Sciences, Iran; 3 Cardiothoracic Department, Tabriz University of Medical Sciences, Tabriz, Iran; 4 Laboratory Department, Tabriz University of Medical Sciences, Tabriz, Iran

**Keywords:** Bacteroides fragilis, surgical site infections, metronidazole, minimum inhibitory concentration, nim gene

## Abstract

**Background:** *Bacteroides fragilis* is the most common anaerobic pathogen isolated from surgical site infections (SSIs). Metronidazole resistance is increasing and the mechanisms of resistance are not clear in some isolates. The aim of the present study was to investigate the metronidazole susceptibility prevalence, and detect *nim* genes in *B. fragilis* isolates from SSIs.

**Methods:** This study included 100 surgery patients with signs and symptoms indicative of SSIs. Syringe aspiration of the infected site was used to collect specimens. All specimens were cultured on BBA (Brucella blood agar), KVLB (kanamycin-vancomycin laked blood), and BBE (Bacteroides bile esculin) agar. The MIC (minimum inhibitory concentration) of metronidazole was determined by the agar dilution method according to the Clinical and Laboratory Standard Institute (CLSI). Then the PCR method was used to determine the presence of the *nim* gene.

**Results:** In the present study, 26 *B. fragilis* were isolated from 100 SSIs specimens. Eight isolates were metronidazole resistant; the metronidazole MIC was 32 µg/mL for 7 isolates and 64 µg/mL for one isolate. All isolates were *nim* gene negative.

**Conclusion:** The emergence of metronidazole-resistant *B. fragilis* limits the application of this drug for treatment and prophylaxis of SSIs. Thus, rapid identification of metronidazole-resistant *B. fragilis* is essential to restrict inappropriate, superfluous administration. In spite of various metronidazole resistance mechanisms other than that depending on the *nim* gene, detection of *nim* by PCR is unsuitable for identifying resistant isolates. Therefore, phenotypic methods are better to screen for and identify metronidazole-resistant *B. fragilis*.

## Zusammenfassung

**Hintergrund:** *Bacteroides fragilis* ist der häufigste bei Surgical Site Infections (SSI) isolierte Anaerobier. Da die Resistenz gegen Metronidazol im Anstieg begriffen ist und die Resistenzmechanismen bei einigen Isolaten unklar sind, sollten die Prävalenz Metronidazol-sensibler Erreger und das Vorkommen von *nim*-Genen bei *B. fragilis*-Isolaten von SSIs untersucht werden.

**Methode:** Es wurden 100 Patienten mit diagnostizierter SSI untersucht. Die mikrobiologischen Proben wurden mittels Nadelaspiration aus dem Infektionsherd gewonnen. Die Kultivierung erfolgte auf Brucella-Blutagar, Kanamycin-Vancomycin-Laked Blutagar und Bacteroides-Galle-Esculin-Agar. Die minimale Hemmkonzentration (MHK) von Metronidazol wurde im Agarverdünnungstest gemäß Clinical and Laboratory Standard Institute (CLSI) bestimmt. Der Nachweis des *nim*-Gens erfolgte mittels PCR.

**Ergebnisse:** In 26 Fällen wurde *B. fragilis* isoliert. Acht Isolate waren gegen Metronidazol resistent (MHK 32 µg/mL bei 7, 64 µg/mL bei einem Isolat). In keinem Isolat war das *nim*-Gen nachweisbar.

**Schlussfolgerung:** Das Vorkommen Metronidazol-resistenter *B. fragilis*-Stämme kann den Erfolg der perioperativen Antibiotikaprophylaxe und ebenso der Therapie infrage stellen. Daher ist die rasche Identifikation Metronidazol-resistenter *B. fragilis*-Stämme wichtig, um eine ungeeignete Verabreichung zu verhindern. In Anbetracht verschiedener *nim*-Gen-unabhängiger Resistenzmechanismen ist der Nachweis des *nim*-Gens mittels PCR zur Identifikation resistenter Isolate ungeeignet. Stattdessen eignen sich phänotypische Methoden zum Screening Metronidazol-resistenter *B. fragilis*-Stämme.

## Introduction

Surgical site infections (SSIs) are mono- and polymicrobial infections caused by anaerobic and aerobic bacteria [1], [2], [3]. *Bacteroides fragilis* is the most important opportunistic anaerobic pathogen isolated from SSIs [4], [5]. Due to its virulence factors such as adhesions, hemagglutinin, polysaccharide capsule, fimbria, and antibiotic resistance, *B. fragilis* is considered to be the most virulent pathogen [6]. Some studies indicate a high prevalence of anaerobic Gram-negative bacilli such as *B. fragilis* in SSIs [7]. Resistance to the most common antimicrobial drugs against Gram-negative anaerobic bacteria, such as β-lactam/β-lactamase inhibitors, fourth-generation fluoroquinolones, carbapenems, metronidazole, clindamycin and other antibiotics, has been reported in *B. fragilis* [8], [9], [10]. The rates of resistance show clinically considerable differences between countries and may also vary from one hospital to another within a country [9]. Metronidazole, a 5-nitroimidazole agent, is the most commonly administrated antibiotic globally for the treatment of infections caused by *B. fragilis* and has been the drug of choice for perioperative surgical prophylaxis in combination with cephalosporins of the 2^nd^ or 3^rd^ generation (e.g. for colorectal surgery) [11]. Metronidazole is administered as an inactive form that activates by reduction of its nitro group under anaerobic condition in the cells. This chemical change gives rise to the active, toxic form, which can break single- and double-strand DNA [12]. Because metronidazole is ineffective against aerobic and facultatively anaerobic bacteria, an additional antibiotic effective against these organisms (e.g., fluoroquinolone or a cephalosporin) is necessary when treating a polymicrobial infection such as SSI [13].

The emergence of metronidazole resistance in *B. fragilis* can lead to a decrease in the efficacy of this drug [14]. Metronidazole-resistant *B. fragilis* were isolated from patients following long-term therapy with this drug [14]. The *B. fragilis* group can develop resistance to this antimicrobial drug by different mechanisms [12]. One of these mechanisms is associated with *nim* A to H genes that encode nitroimidazole reductase, which transforms 4- or 5-nitroimidazole to 4- or 5-aminoimidazole, the nontoxic derivative [8], [12], [14]. Other mechanisms include overexpression of the multidrug efflux pump, overexpression of RecA protein, and deficiency of the ferrous iron transporter FeoAB [11], [12], [15]. 

The aim of this study was to assess the prevalence of *B. fragilis* in SSIs, its susceptibility to metronidazole and the presence of the *nim* gene in these isolates.

## Materials and methods

### Patients

This descriptive cross-sectional study was conducted at Imam Reza Hospital in Tabriz, Iran. The study was carried out for a period of 10 months from October 2013 to July 2014 and involved 100 hospitalized patients. SSIs are wound infections that occur within 30 days after the surgery. The patients were selected according to isolation of pathogenic microorganisms and clinical signs of SSIs (inflammation, irritation, fever, and discharge). For sampling, first the skin was disinfected by povidone iodine, and then specimens were collected by syringe aspiration of material deep within the infected site. The syringe was immediately sealed and transported to the laboratory within 20 min. Inoculation generally took place at the latest 1 hour after collection.

### Bacterial isolation

The cytology assessment and detection of bacteria in specimens was performed by the Gram stain smear. Pre-reduced media including vitamin K-enriched Brucella blood agar, kanamycin-vancomycin laked blood agar (KVLB, the basal medium is Brucella agar; Fluka Chemie AG, Buchs, Switzerland) and Bacteroides bile esculin agar (BBE, Himedia Laboratories Pvt. Ltd, India) were inoculated for the isolation of *B. fragilis* [16], [17]. The plates were incubated under 80% N_2_, 10% CO_2_, 10% H_2_ and 0% O_2_ in an anaerobic jar using the Anoxomat system (MART microbiology B.V., the Netherlands), and these plates were examined at 48, 72, and 96 h. The aerotolerance test, biochemical tests (such as reaction in the BBE agar, catalase production, indole, and sugar fermentation) and the MAST ID MID8 ANAEROBE ID RING (MAST CO.) were used to identify *B. fragilis* isolates [17], [18].

### Determination of metronidazole MIC

MIC was determined by the agar dilution method on Brucella agar (supplemented with 5% sheep blood, vitamin K (1 µg/mL) and hemin (5 µg/mL), and 1×10^4^ CFU were inoculated on agar according to the recommendation of the CLSI [19]. Ten dilution steps ranging from 0.25 to 128 µg/ml (0.25, 0.5, 1, 2, 4, 8, 16, 32, 64, and 128 µg/mL) were tested for metronidazole susceptibility testing. The plates were incubated at 37°C and read at 48 h after incubation in anaerobic conditions. The lowest antibiotic concentration at which growth of bacteria was inhibited was determined as the MIC. The results were interpreted according to the CLSI guideline [19]. 

### DNA extraction and detection of nim genes

One loopful of cultured B. fragilis was suspended in 400 µL of TE buffer (10 mM Tris-HCL, 1 mM EDTA, PH 8.0) and kept at 80°C for 30 min to kill the bacteria. DNA was extracted by CTAB, SDS, and proteinase K. After sedimentation with isopropanol and washing with ethanol 70%, extracted DNA was solved in 100 µL TE buffer [20].

For the PCR reaction, specific primers of 458-bp fragment NIM-3 (5'-ATG TTC AGA GAA ATG CGG CGT AAG CG-3') and NIM-5 (5'-GCT TCC TTG CCT GTC ATG TGC TC-3') were used [20]. Each PCR assay was performed in 25 µL of reaction mixture containing 2 µL of DNA sample, 2 µL of each primer, 1.25 µL of MgCl_2_ (50 mM), 0.5 µL d NTP (10 mM), 2.5 µL of PCR Buffer (10X), 0.5 µL of bovine serum albumin (10 mg/mL), 1 µL of *Taq* polymerase (2.5 u/µL) and 13.25 µL of distilled water [20]. Target DNA was amplified using a thermal cycler (Gradient Eppendorf). After an initial denaturation step at 94°C for 10 min, the reaction mixture was subjected to 32 cycles of amplification consisting of denaturation at 94°C for 30 s, annealing at 62°C for 1 min, extension at 72°C for 1 min, and a final extension step at 72°C for 10 min [20]. The PCR products were analyzed by electrophoresis on 1.2% agarose gels in TBE buffer (89 mM Tris base, 89 mM boronic acid, 2 mM Na_2_, EDTA, pH 8.25). The agarose gel was stained with 0.5 µg/mL ethidium bromide and visualized under UV light. A 100 bp ladder was used as molecular size marker [20]. 

## Results

A total of 100 SSI specimens were processed; *B. fragilis* was isolated from 26 of them (in 14 male and 12 female patients) (Table 1 (Tab. 1)). All *B. fragilis* was isolated from polymicrobial infections. Overall, the greatest number of *B. fragilis* isolates were recovered from gastrointestinal tracts (23 cases), followed by respiratory tracts (2 cases) and orthopedic (1 case) surgery procedure. The metronidazole MIC values for these isolates ranged from 0.5 to 64 µg/mL. According to the agar dilution method, 8 (30.76%) of 26 isolates were found to be metronidazole resistant. The metronidazole MIC for 7 isolates was 32 µg/mL and for one isolate 64 µg/mL. When these isolates were subjected to PCR to screen for the presence of the nim gene, all isolates were negative. The components of PCR reactions were cross checked for the confidence of action and repeated 3 times. Interestingly, all of the metronidazole-resistant isolates were recovered from patients receiving metronidazole as a prophylaxis before surgery. On the other hand, there was a history of metronidazole use in metronidazole-susceptible *B. fragilis* as well.

## Discussion

SSIs are largely polymicrobial in nature, involving both aerobic and anaerobic bacteria [4]. These bacterial pathogens cause infection and delay healing. Anaerobic bacterial pathogens remain a significant cause of morbidity and mortality in surgical patients [4]. In some studies, *B. fragilis* was the most common anaerobic pathogen isolated from SSIs [5], [7]. 

In our study, this organism was isolated from 26% of the patient sample. By way of comparison, Brook [5] isolated *B. fragilis* from 36% of patients, while Giacometti [1] isolated *B. fragilis* from 1.3% of patients. Results of a study by Wollcot et al. [7] which assessed microbial diversity in SSIs using molecular methods indicate the high prevalence of anaerobic Gram-negative rods including *B. fragilis* in these infections. Since anaerobic bacteria require certain conditions for specimen collection, transport and culture media for *in vitro* growth, some studies on SSIs may underestimate the prevalence of anaerobic bacteria [4], [7]. 

In recent years, decreasing susceptibility has been found to a wide range of antimicrobial agents, including metronidazole among *B. fragilis* strains, which may limit their therapeutic efficiency in treating infections caused by this organism during the course of metronidazole therapy [14]. In 8 patients (30.8%) of this study, we isolated metronidazole-resistant *B. fragilis* by the agar dilution method, but *nim* genes were not detectable in these isolates. In previous studies, metronidazole-resistant *B. fragilis* strains were isolated from 3.9% (Spain, 2010) to 15% (UK, 2004) of patients examined [21], [22]. 

This study was nosocomial, and *B. fragilis* were isolated from hospitalized patients with metronidazole administration for prophylaxis and/or treatment of SSIs. The literature describes an association between isolation of metronidazole-resistant *B. fragilis* and long-term metronidazole therapy [8], [14], [21]. Increasing numbers of clinical metronidazole-resistant *B. fragilis* that do not possess *nimA-H* genes are being reported. Resistance to metronidazole could be induced in *nim*-negative strains by exposure to sub-MICs of metronidazole; the mechanisms behind the increased MICs are not obvious. However, it is clear that there is also a non-*nim*-based mechanism of resistance to metronidazole [11], [13]. In addition, exposure to sub-inhibitory concentrations of metronidazole may enhance the pathogenicity of the non-susceptible strains of *B. fragilis* [23], [24]. 

In our study, no correlation was found between resistance to metronidazole and *nim* gene presence. In one study [14], metronidazole-resistant *B. fragilis* were identified from 55% of clinical isolates, while the *nim* gene was detected from 38.9% isolates. Some studies indicated no correlation between Nim protein levels and metronidazole MIC, and no evidence was found that *nim* protects *B. fragilis* from metronidazole. Indeed, the contribution of the *nim* gene to high metronidazole MICs seen in clinical resistance is unclear [25]. 

In our study, resistance to metronidazole may be due to non-*nim*-based mechanisms, such as overexpression of the multidrug efflux pump, overexpression of Rec A, deficiency of feo AB, or some other, as yet unknown mechanism. Regardless of the mechanism, development of metronidazole-resistant *B. fragilis* may lead to treatment and prophylaxis failure during the course of metronidazole therapy in patients with SSIs caused by *B. fragilis*. Thus, rapid identification of metronidazole-resistant isolates is essential for early initiation of appropriate antimicrobial therapy and to limit the inappropriate use of antimicrobial agents. 

## Notes

### Acknowledgments

This research was supported by a grant from the Infectious and Tropical Disease Research Center of Tabriz University of Medical Sciences (TBZMED). The manuscript was written based on a dataset of the M.Sc. thesis of Mohammad Yousef Memar registered at Tabriz University of Medical Sciences. The authors would like to thank the staff of Imam Reza surgical wards and microbiology department for their help.

### Compliance with ethical standards

The Ethics Commission of Tabriz University of Medical Sciences approved this study (Number: 5/4/6256). All procedures performed in studies involving human participants were in accordance with the ethical standards of the institution.

### Competing interests

The authors declare that they have no competing interests.

## Figures and Tables

**Table 1:**
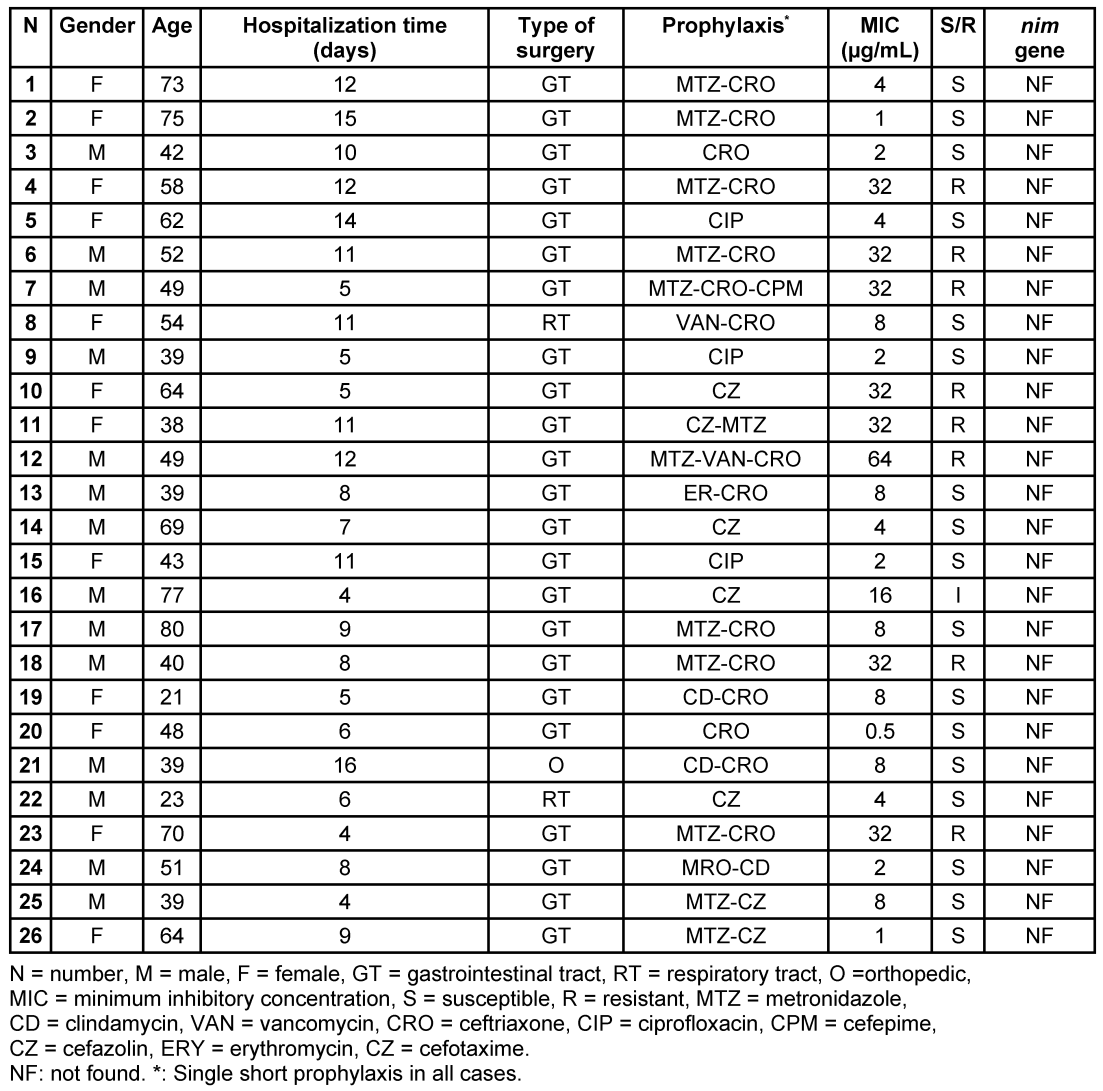
Clinical characteristics of patients with *B. fragilis* infection
